# Identification of prognostic cancer-associated fibroblast markers in luminal breast cancer using weighted gene co-expression network analysis

**DOI:** 10.3389/fonc.2023.1191660

**Published:** 2023-05-03

**Authors:** An Xu, Xiang-Nan Xu, Zhou Luo, Xiao Huang, Rong-Quan Gong, De-Yuan Fu

**Affiliations:** ^1^ Medical College of Yangzhou University, Yangzhou, Jiangsu, China; ^2^ Department of Thyroid and Breast Surgery, Northern Jiangsu People’s Hospital, Yangzhou, Jiangsu, China

**Keywords:** luminal breast cancer (LBC), cancer-associated fibroblasts (CAFs), weighted gene co-expression network analysis (WGCNA), prognostic CAF markers, anti-CAF therapeutic approach

## Abstract

**Background:**

Cancer-associated fibroblasts (CAFs) play a pivotal role in cancer progression and are known to mediate endocrine and chemotherapy resistance through paracrine signaling. Additionally, they directly influence the expression and growth dependence of ER in Luminal breast cancer (LBC). This study aims to investigate stromal CAF-related factors and develop a CAF-related classifier to predict the prognosis and therapeutic outcomes in LBC.

**Methods:**

The Cancer Genome Atlas (TCGA) and Gene Expression Omnibus (GEO) databases were utilized to obtain mRNA expression and clinical information from 694 and 101 LBC samples, respectively. CAF infiltrations were determined by estimating the proportion of immune and cancer cells (EPIC) method, while stromal scores were calculated using the Estimation of STromal and Immune cells in MAlignant Tumors using Expression data (ESTIMATE) algorithm. Weighted gene co-expression network analysis (WGCNA) was used to identify stromal CAF-related genes. A CAF risk signature was developed through univariate and least absolute shrinkage and selection operator method (LASSO) Cox regression model. The Spearman test was used to evaluate the correlation between CAF risk score, CAF markers, and CAF infiltrations estimated through EPIC, xCell, microenvironment cell populations-counter (MCP-counter), and Tumor Immune Dysfunction and Exclusion (TIDE) algorithms. The TIDE algorithm was further utilized to assess the response to immunotherapy. Additionally, Gene set enrichment analysis (GSEA) was applied to elucidate the molecular mechanisms underlying the findings.

**Results:**

We constructed a 5-gene prognostic model consisting of RIN2, THBS1, IL1R1, RAB31, and COL11A1 for CAF. Using the median CAF risk score as the cutoff, we classified LBC patients into high- and low-CAF-risk groups and found that those in the high-risk group had a significantly worse prognosis. Spearman correlation analyses demonstrated a strong positive correlation between the CAF risk score and stromal and CAF infiltrations, with the five model genes showing positive correlations with CAF markers. In addition, the TIDE analysis revealed that high-CAF-risk patients were less likely to respond to immunotherapy. Gene set enrichment analysis (GSEA) identified significant enrichment of ECM receptor interaction, regulation of actin cytoskeleton, epithelial-mesenchymal transition (EMT), and TGF-β signaling pathway gene sets in the high-CAF-risk group patients.

**Conclusion:**

The five-gene prognostic CAF signature presented in this study was not only reliable for predicting prognosis in LBC patients, but it was also effective in estimating clinical immunotherapy response. These findings have significant clinical implications, as the signature may guide tailored anti-CAF therapy in combination with immunotherapy for LBC patients.

## 1 Introduction

Breast cancer (BC) is the most prevalent cancer among women worldwide and the second leading cause of cancer deaths (1, 2). While the current standard treatment for breast cancer has greatly improved survival, it remains a public health issue on a global scale (3). LBC is a subtype of breast cancer, including Luminal A and Luminal B, characterized by the presence of estrogen and/or progesterone receptors on the surface of cancer cells (4). Although LBC has the best prognosis among breast cancer subtypes, approximately 20-40% of LBCs eventually develop distant metastases, with half occurring 5 years or later after the diagnosis of the primary tumor (5). The tumor microenvironment (TME) in breast cancer comprises local factors, cancer cells, immune cells and stromal cells of the local and distant tissues (6, 7). Accumulating evidence indicated that the interaction between LBC cells and their microenvironment plays important roles in tumor proliferation, propagation and response to therapies (8–10).

CAFs are important components of the tumor microenvironment (TME) and are widely distributed in tumor stroma (11, 12). They play a crucial role in promoting tumor growth through direct effects on tumor cells and various interactions with receptors and ligands (13, 14). Moreover, they indirectly stimulate tumor growth and migration by releasing growth factors, cytokines, and exosomes, inducing metabolic reprogramming and anti-tumor resistance, and suppressing the immune system (15–17). Additionally, CAFs help to create a physical barrier through the deposition and reorganization of the extracellular matrix, which supports tumor cell invasion and restrains antitumor leukocyte infiltration, leading to tumor progression, immune evasion, and therapy resistance (18).

Studies have shown that CAFs can affect the response of LBC to hormone therapy, a common treatment, by altering the expression of estrogen receptors on cancer cells (19–21). Targeting CAFs can be achieved through various methods such as influencing secreted factors and signaling pathways, inducing a quiescent state in CAFs or targeting CAF-derived cells (18, 22). For example, losartan, an angiotensin receptor blocker, can convert myofibroblast CAFs into a quiescent state and enhance immune cell activity, thus improving the response of breast cancer cells to immune checkpoint blockers (23). In addition, blocking CD10 and GPR77 with neutralizing antibodies can decrease tumor growth and increase chemotherapy sensitivity in breast cancer models (24). Therefore, identifying common markers of CAFs can lead to the discovery of more specific markers and therapeutic targets for LBC.

Weighted gene co-expression network analysis (WGCNA) is a powerful bioinformatics algorithm that can identify highly and coordinately expressed genes and group them into gene modules to explore their relationships with a phenotype of interest (25). WGCNA has been previously used to identify cancer-associated fibroblast (CAF) markers (26–29). However, there has been no WGCNA analysis conducted on CAF and stromal infiltrations in LBC.

In this study, we employed WGCNA simultaneously on two transcriptome datasets from TCGA and GEO databases. We identified hub modules that were most correlated with stromal CAF infiltrations. Using univariate and Least Absolute Shrinkage and Selection Operator (LASSO) Cox regression analyses, we identified RIN2, THBS1, IL1R1, RAB31, and COL11A1 as prognostic CAF markers. We then constructed a five-gene CAF signature that could predict prognosis and therapeutic responses in LBC. Our findings suggest that the CAF model could be a promising anti-CAF therapeutic approach for LBC.

## 2 Materials and methods

### 2.1 The collection and preparation of data

The transcript per million (TPM) format RNA-seq data and clinical information relevant to Breast Invasive Carcinoma (TCGA-BRCA) samples were obtained from TCGA datasets (https://portal.gdc.cancer.gov/). The gene expression profiling dataset (GSE47994) was obtained from the Gene Expression Omnibus (https://www.ncbi.nlm.nih.gov/) database (30). We then screened a total of 694 LBC patients from the TCGA-BRCA dataset and 101 LBC patients from the GSE47994 dataset who had prognostic data available, with follow-up times exceeding 365 days.

### 2.2 The estimation of CAF infiltration and the calculation of stromal score

Four methods were utilized to estimate the abundance of CAFs, including the Estimate the Proportion of Immune and Cancer cells (EPIC) algorithm based on cell-type deconvolution using constrained least square optimization (31), the xCell algorithm based on gene signature enrichment (32), the microenvironment cell populations-counter (MCP-counter) based on marker gene expressions (33), and the Tumor Immune Dysfunction and Exclusion (TIDE) algorithms, which also can predict anti-PD1 and anti-CTLA4 responses in tumor patients (34). The first three methods were executed *via* the deconvolute() function of the immunedeconv R package (version 2.0.3) (35), while the TIDE method was implemented through http://tide.dfci.harvard.edu/. Additionally, the Estimation of STromal and Immune cells in MAlignant Tumor tissues using Expression data (ESTIMATE) algorithm was utilized to calculate the stromal score, which indicates the level of stromal infiltration in each tumor sample, through the estimate R package (version 1.0.13) (36).

### 2.3 The creation of CAF and stromal co-expression networks

The WGCNA R package (version 1.72) was utilized to construct co-expression networks and identify hub genes that target cancer-associated fibroblast (CAF) infiltrations and stromal scores (25). The input genes for network construction were selected based on the median absolute deviation (MAD), with the top 5,000 genes selected for both the TCGA-BRCA and GSE47994 cohorts. The Pearson’s correlation similarity matrix was calculated between each pair of genes (s_ij_, where ij represents the gene pairs) and raised to a soft-thresholding power β ( ${\text{S}}_{\text{ij}}^{\text{β}}$ ), based on the scale-free topology network criterion. The adjacency matrix was then clustered using the topological overlap measure (TOM) and dissimilarity (1-TOM) between genes, and a dynamic tree cut algorithm was applied to the dendrogram to identify gene modules with a minimum of 30 genes in each module. The first principal component of each module’s expression was summarized as a module eigengene (ME), and the Pearson’s correlations between MEs and EPIC-quantified CAF infiltrations, as well as the stromal score, were assessed to identify the most correlated module for further analysis. Hub genes were then identified by overlapping the most correlated module genes between the TCGA-BRCA and GSE47994 cohorts.

### 2.4 The analysis of gene ontology and the kyoto encyclopedia of genes and genomes

The clusterProfiler R package (version 3.14.3) was utilized to analyze the hub genes’ biological functions, which included biological processes (BPs), molecular functions (MFs), and cellular components (CCs), as well as pathways according to GO and KEGG databases (37). p < 0.05 was considered statistically significant.

### 2.5 The creation and verification of a predictive algorithm

For CAF risk model construction, 694 LBC cases from TCGA-BRCA were selected based on their large sample size. The validation cohort consisted of 101 LBC cases from GSE47994 cohort. Univariate Cox regression analysis was performed to identify prognostic stromal CAF hub genes for overall survival (OS). Genes with p < 0.05 were selected for LASSO Cox regression analysis with 1,000 iterations using glmnet R package to reduce the number of genes (38). Then, the CAF risk model was constructed as follows: CAF risk score = ∑ (β_i_ * Exp_i_), where β_i_ is the LASSO coefficient of *i*th gene and Exp_i_ is the expression value of *i*th gene. Using the median CAF risk score of the training cohort as the threshold, LBC patients from both cohorts were classified into high- and low-CAF-risk groups and the OS difference between the two groups was estimated using Kaplan–Meier curves and the log-rank test.

### 2.6 The collection of CAF markers and the analysis of their correlation

We collected CAF specific and nonspecific markers from published literature (39, 40). To verify the reliability of our CAF model markers in LBC, we examined the Spearman’s correlations between the CAF risk score and the stromal score, as well as various CAF infiltration estimates (EPIC, xCell, MCP-counter, and TIDE). We also analyzed the correlations between CAF model genes and published CAF markers in both TCGA-BRCA and GSE47994 cohorts.

### 2.7 The prediction of chemotherapy and immunotherapy responses

Using the Genomics of Drug Sensitivity in Cancer [GDSC (https://www.cancerrxgene.org/)] database (41), half-maximal inhibitory concentration (IC50) values of common drugs (bleomycin, lapatinib, paclitaxel, camptothecin, cisplatin, docetaxel, methotrexate, and sunitinib) in each LBC sample were estimated based on the transcriptome data by ridge regression with ten-fold cross-validation in pRRophetic R package (version 0.5) (42). The TIDE (http://tide.dfci.harvard.edu/) online algorithm was then adopted for immune checkpoint blockade therapy response predictions (34). The chi-squared test was used to examine differences in response rates between high- and low-CAF-risk groups. The predictive efficacy of the CAF risk signature was evaluated by ROC curves and area under the curve (AUC) values.

### 2.8 Analysis of enrichment

To explore the enriched hallmark and KEGG pathway gene sets between high- and low-CAF-risk groups in GSE47994, gene set enrichment analysis (GSEA) was performed using the enrichplot and clusterProfiler R packages. The gene sets used were derived from the Molecular Signatures Database (MSigDB), specifically the “c2.cp.kegg.v7.4.symbols” and “h.all.v7.4.symbols” gene sets (43). Additionally, the enrichment scores of ECM receptor interaction, regulation of actin cytoskeleton, and TGF-β signaling pathway hallmark gene sets were calculated using ssGSEA (44). Finally, Spearman’s correlation analysis was performed to assess the correlation between the CAF risk score and gene set enrichment scores.

### 2.9 The verification of results using the cancer cell line encyclopedia and human protein atlas databases

To validate the findings at the cellular level, mRNA expressions of the identified markers in 38 fibroblasts and 51 BC cell lines were downloaded from the Cancer Cell Line Encyclopedia [CCLE (https://portals.broadinstitute.org/ccle)] database (45). Expression patterns of these markers in fibroblasts and CRC cell lines were examined using heat maps and Wilcoxon tests. Additionally, immunohistochemical (IHC) staining images of these markers in LBC tissues were downloaded from the Human Protein Atlas [HPA (https://www.proteinatlas.org/)] online database (46).

### 2.10 Statistical analysis of the data

R software (version 4.2.2; https://www.r-project.org/) was used for all statistical analyses. The median CAF risk score was used as the cutoff value for each cohort to divide LBC patients into high- and low-CAF-risk subgroups. Pairwise comparisons were performed using the Wilcoxon test. Pearson correlation coefficient analysis was performed to evaluate the correlation between genes. Overall survival comparisons were made using the Kaplan-Meier curve with the log-rank test, which were adopted *via* the survival and survminer R packages. p < 0.05 was considered statistically significant.

## 3 Results

### 3.1 The prognostic value of CAF infiltrations and stromal scores in LBC patients

**Figure 1:**
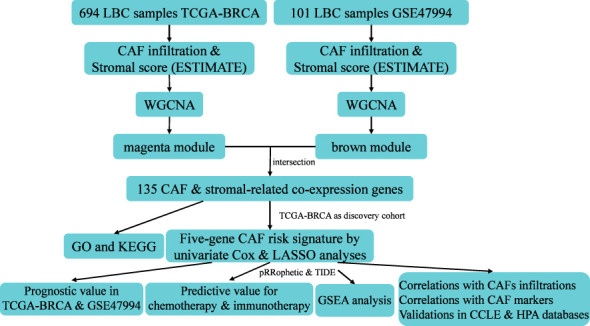
Work flow of this study.

**Figure 2:**
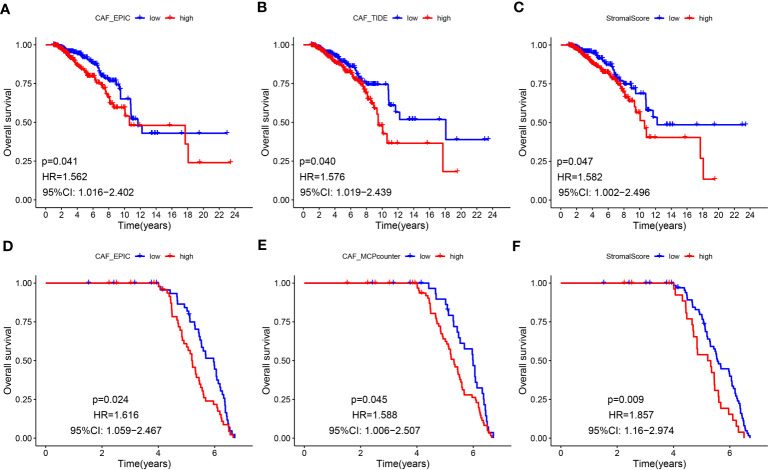
**(A-F)** Kaplan–Meier analyses of LBC patients. Multiple higher CAF infiltrations and stromal scores were significantly associated with worse overall survival in TCGA-BRCA **(A–C)** and GSE47994 **(D–F)**.

The flowchart of this research is displayed in **Figure 1**. CAF infiltrations were predicted by multiple methods, including EPIC, xCell, MCP-counter, and TIDE. The stromal score was calculated by the estimate algorithm. Their prognostic values on overall survival (OS) were evaluated *via* log-rank tests. Kaplan-Meier curves indicated that multiple higher CAF infiltrations and stromal scores were significantly associated with poorer OS in LBC patients. CAF_EPIC, CAF_TIDE, and stromal scores were significantly associated with poorer OS in TCGA-BRCA (**Figures 2A–C**), and CAF_EPIC, CAF_mcp-counter, and stromal scores were significantly associated with poorer OS in GSE47994 (**Figures 2D–F**), which highlights the importance of further studies exploring CAF and stromal-associated genes in LBC. In this study, the EPIC-estimated CAF abundances and stromal scores were summarized as phenotype data for subsequent analysis, and the data from the other three estimated CAF infiltrations were used for external validation of the identified CAF model.

### 3.2 Co-expression network analysis of CAF and stromal scores in two LBC datasets

**Figure 3:**
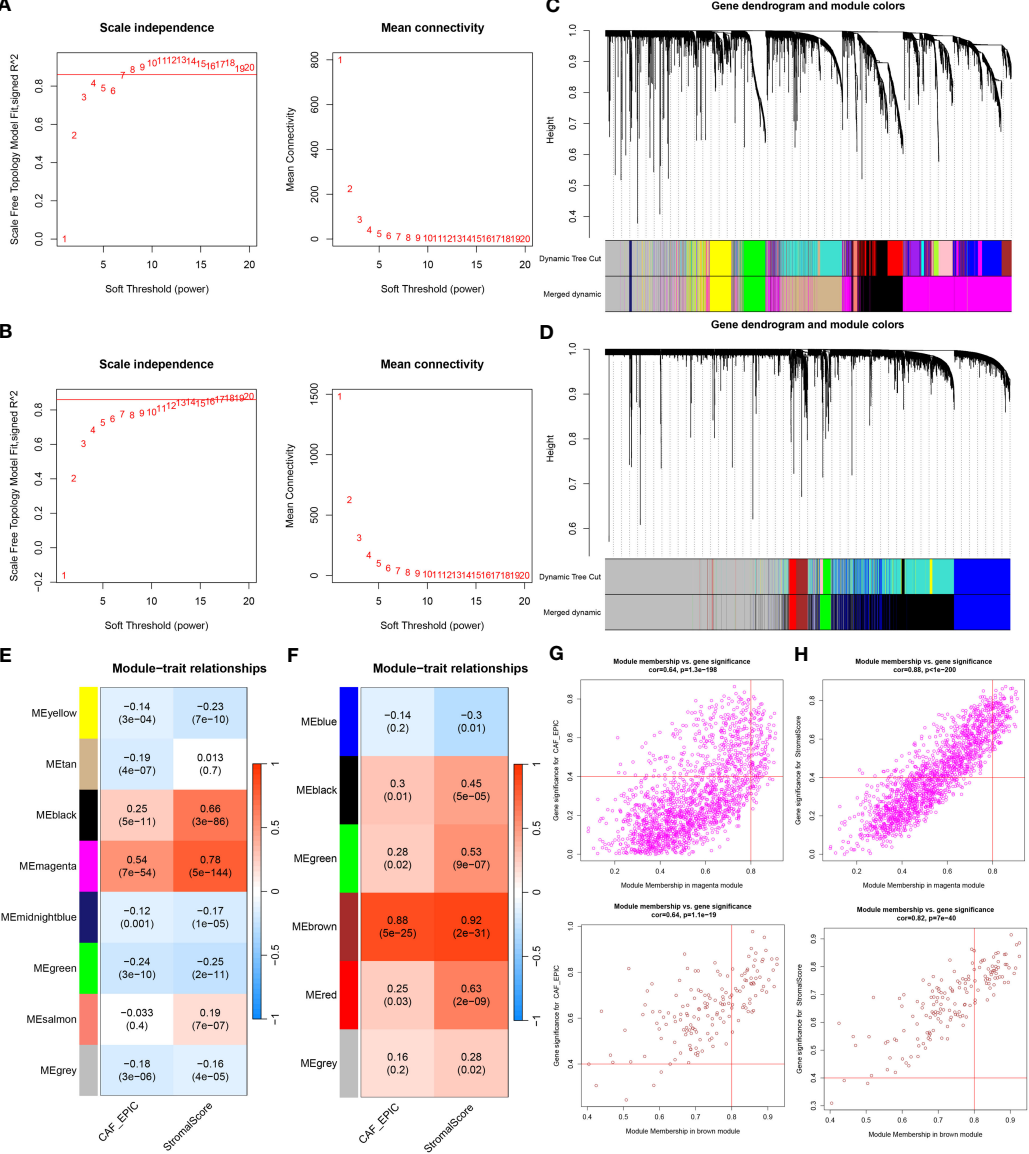
**(A, B)** Co-expression network constructed by WGCNA. The soft-thresholding power (β) of 7 and 17 was, respectively, selected based on the scale-free topology criterion in TCGA-BRCA **(A)** and GSE47994 **(B)**. **(C, D)**. Clustering dendrograms showing genes with similar expression patterns were clustered into co-expression modules in TCGA-BRCA **(C)** and GSE47994 **(D)**. The gray module indicates that genes were not assigned to any module. **(E, F)** Module-trait relationships revealing the correlations between each gene module eigengene and phenotype in TCGA-BRCA **(E)** and GSE47994 **(F)**. **(G, H)** Scatter plots of the module membership (MM) and gene significance (GS) of each gene in the magenta module of TCGA-BRCA **(G)** and the brown module of GSE47994 **(H)**. The horizontal axis is the correlation between the gene and co-expression module, and the vertical axis is the correlation between the gene and phenotype.

WGCNA analysis was conducted on both TCGA-BRCA and GSE47994 datasets. To build a scale-free topology network, we estimated the soft threshold power (β) of 7 in TCGA-BRCA (scale-free R2 = 0.86) (**Figure 3A**) and 17 in GSE47994 (scale-free R2 = 0.86) (**Figure 3B**). In TCGA-BRCA, the hierarchical clustering tree identified 8 co-expression models (**Figure 3C**), and the magenta module exhibited the strongest positive correlation with the CAF proportion (Cor = 0.54, P = 7e-54) and stromal score (Cor = 0.78, P = 5e-144) (**Figure 3E**). In GSE47994, the dynamic hybrid cutting clustered 6 co-expression models (**Figure 3D**), with the brown module showing the strongest positive correlation with the CAF proportion (Cor = 0.88, P = 5e-25) and stromal score (Cor = 0.92, P = 2e-31) (**Figure 3F**). Therefore, we focused on these two modules for further investigations. A total of 1718 and 158 genes were included in the magenta and brown modules, respectively. In the magenta module, scatter plots indicated strong correlations between MM and GS for CAF (Cor = 0.64, p =1.3e-198) and stromal scores (Cor = 0.88, p < 1e-200) (**Figure 3G**). Similarly, in the brown module, strong correlations were observed between MM and GS for CAF (Cor = 0.64, P = 1.1e-19) and stromal scores (Cor = 0.82, p = 7e-40) (**Figure 3H**). Consequently, we selected 1718 genes from the TCGA-BRCA magenta module and 158 genes from the GSE47994 brown module as highly associated with CAF and stromal scores.

### 3.3 Functional analyses of CAF-related genes

**Figure 4:**
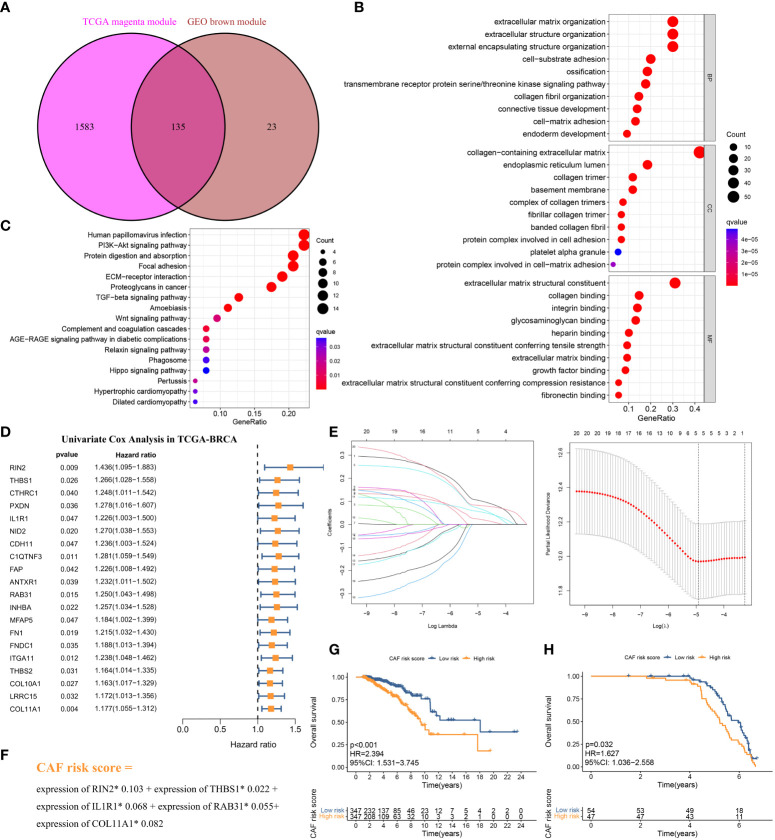
**(A)** The intersection of TCGA-BRCA magenta and GSE47994 brown module genes was presented in the Venn diagram. **(B, C)** GO analyses of the enriched biological process (BP), cellular component (CC), and molecular function (MF) terms **(B)** and KEGG pathway analysis **(C)** of the 135 genes. **(D)** Univariate Cox analysis for the screening of overall survival-associated genes in TCGA-BRCA. **(E)** Coefficient profiles of least absolute shrinkage and selection operator (LASSO) Cox regression analysis, and the adjustment parameter (lambda) was calculated based on the partial likelihood deviance with ten-fold cross validation. **(F)** Formulation of the CAF risk model. **(G, H)** Kaplan–Meier analyses identified gastric cancer patients in the high–CAF-risk group which exhibited worse overall survival in both TCGA-BRCA **(G)** and GSE47994 **(H)** cohorts.

The above CAF-related genes were overlapped and screened to 135 hub genes (**Figure 4A**). These genes were subjected to Gene Ontology (GO) and Kyoto Encyclopedia of Genes and Genomes (KEGG) analyses. The major enriched GO terms were related to extracellular matrix organization, extracellular structure organization, external encapsulating structure organization (BP), collagen-containing extracellular matrix and endoplasmic reticulum lumen (CC), and extracellular matrix structural constituents and collagen binding (MF) (**Figure 4B**). The main enriched KEGG pathways were human papillomavirus infection, the PI3K-Akt signaling pathway, and protein digestion and absorption (**Figure 4C**).

### 3.4 Construction a risk model based on stromal CAF

The 694 LBC samples from TCGA- BRCA were used as the training cohort owing to the larger sample size, and 101 GSE47994 samples were used as the validation group. By performing univariate Cox regression analysis of the 135 common hub genes, 20 OS-related genes with p < 0.05 were screened out and subjected to the following LASSO Cox regression analysis (**Figures 4D, E**). Five genes were finally identified for the CAF risk model construction: CAF risk score = expression of RIN2* 0.103 + expression of THBS1* 0.022 + expression of IL1R1* 0.068 + expression of RAB31* 0.055 + expression of COL11A1* 0.082 (**Figure 4F**). The median CAF risk score of the training cohort was 1.85, which was used as the cut-off value to classify LBC patients from each cohort into high- and low-CAF-risk groups. LBC patients in each cohort were divided into high– and low–CAF-risk groups with the median risk score as the cutoff value. Kaplan–Meier curves revealed that LBC patients in the high–CAF-risk group experienced worse OS than those in the low–CAF-risk group in both TCGA- BRCA (HR = 2.394, 95%CI: 1.531–3.745, log-rank p < 0.001) (**Figure 4G**) and GSE47994 (HR = 1.627, 95%CI: 1. 036−2.558, log-rank p = 0.032) (**Figure 4H**). These results indicated CAF and stromal-related signature genes were crucial prognostic markers in LBC.

### 3.5 Validation of the CAF risk score and the five-gene signature as indicators of CAF infiltrations

**Figure 5:**
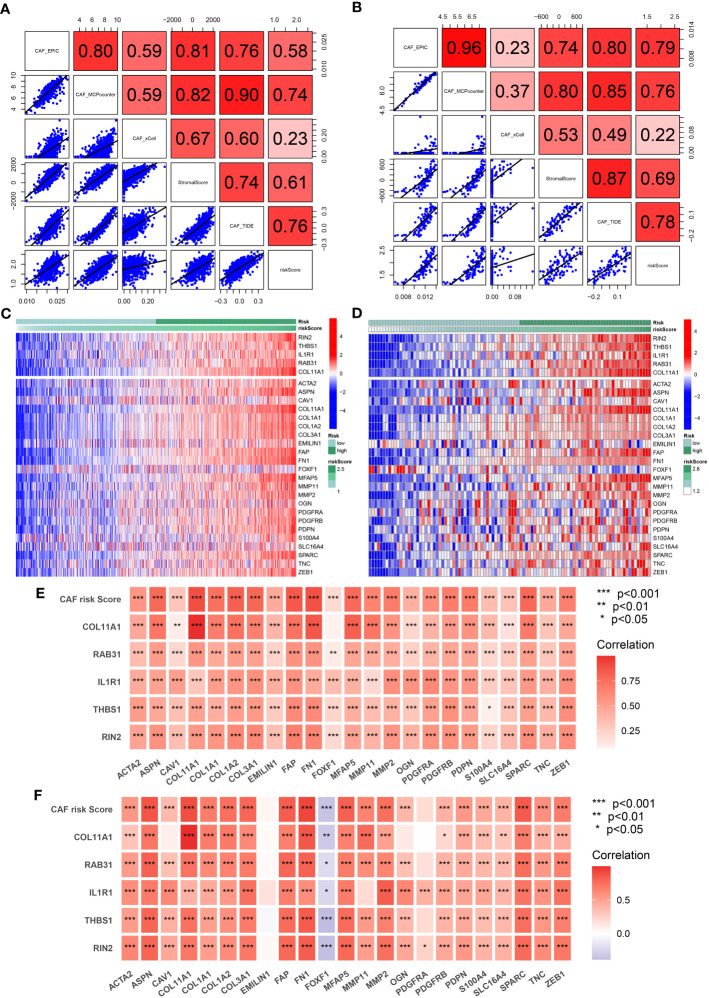
**(A, B)** Spearman’s correlation analyses revealing the CAF risk score was strongly and positively correlated with stromal scores and multi-estimated CAF infiltrations in TCGA-BRCA **(A)** and GSE47994 **(B)** cohorts. **(C, D)** The heat map revealing the expression patterns of CAF markers identified five CAF genes with the CAF risk score in TCGA-BRCA **(C)** and GSE47994 **(D)** cohorts. **(E, F)** The CAF risk score and five signature genes were positively correlated with literature that reported CAF markers in TCGA-BRCA **(E)** and GSE47994 **(F)** cohorts.

To evaluate the robustness of the CAF model as an indicator of CAF infiltrations, Spearman’s correlation analyses were performed between the CAF risk score and stromal score as well as CAF abundances predicted by EPIC and three other methods: xCell, MCP-counter, and TIDE. The CAF risk score was strongly and positively correlated with multi-estimated CAF infiltrations and the stromal score in both TCGA-BRCA (**Figure 5A**) and GSE47994 (**Figure 5B**) cohorts. These results confirmed that the CAF risk score was a reliable predictor of CAF infiltrations. To further validate the correlation of the expression levels of the five genes with CAFs, their expression levels were compared with a set of collected CAF markers in both TCGA-BRCA (**Figures 5C, E**) and GSE47994 (**Figures 5D, F**) cohorts. A high and positive correlation was observed between the expression levels of the five genes and most of the CAF markers in both cohorts. These results demonstrated that the five genes were representative of CAFs.

### 3.6 Chemotherapy and immunotherapy responses across CAF-risk groups

**Figure 6:**
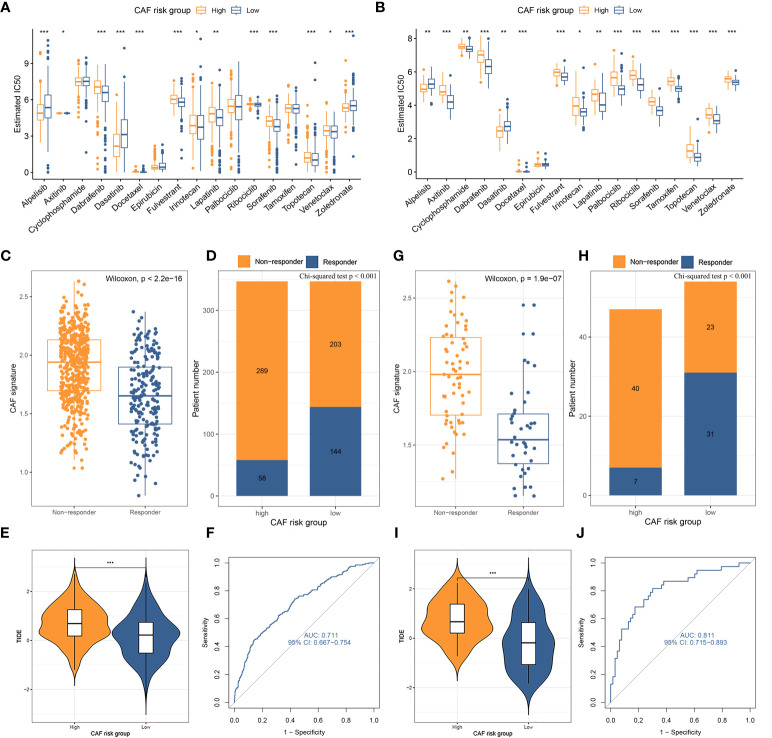
**(A, B)** Box plots comparing IC50 values of several chemotherapy drugs between high– and low–CAF-risk groups in TCGA-BRCA **(A)** and GSE47994 **(B)** cohorts. **(C–J)** TIDE immunotherapy prediction analyses. **(C, G)** The CAF risk score between TIDE-predicted immunotherapy-responders and non-responders in TCGA-BRCA **(C)** and GSE47994 **(G)**; **(D, H)** Distributions of responders and non-responders in high– and low– CAF-risk groups in TCGA-BRCA **(D)** and GSE47994 **(H)**; **(E, I)** Distributions of TIDE scores in high– and low– CAF-risk groups in TCGA-BRCA **(E)** and GSE47994 **(I)**; **(F, J)** ROC curves of the CAF risk score in predicting immunotherapy responses in TCGA-BRCA **(F)** and GSE47994 **(J)**. ^∗^p < 0.05, ^∗∗^p < 0.01, ^∗∗∗^p < 0.001.

The standard treatment for LBC patients involves radical surgery followed by adjuvant chemotherapy and endocrine therapy (47). IC50 values for multiple anti-tumor drugs, including those used in breast cancer treatment, were estimated using the GDSC database for both the TCGA-BRCA (**Figure 6A**) and GSE47994 (**Figure 6B**) cohorts. Wilcoxon analyses indicated significant differences in IC50 values between high- and low-CAF-risk LBC patients. As for commonly used chemotherapy drugs for breast cancer, although not both datasets showed statistical significance, the results indicated that high-CAF-risk patients were sensitive to Alpelisib and Epirubicin, while low-CAF-risk patients were sensitive to Docetaxel, Fulvestrant, Lapatinib, Palbociclib, Ribociclib and Tamoxifen. However, Cyclophosphamide and Zoledronic acid exhibited different trends between the two datasets. In addition, the results from both datasets showed that high-CAF-risk patients were insensitive to several other drugs, including Axitinib, Dabrafenib, Irinotecan, Sorafenib, Topotecan, and Venetoclax. This suggests that higher CAF risk scores are more likely to induce resistance to these drugs in breast cancer patients. Immunotherapy is among the most important advances in recent oncology, particularly for triple-negative and HER-2-positive breast cancer (48). Trials are also underway to assess the efficacy of immune checkpoint inhibitors such as pembrolizumab for Luminal breast cancer (49–51). To evaluate the CAF risk score as an immunotherapy predictor for LBC patients, the TIDE method was used. In TCGA-BRCA, the non-responder subgroup (n = 492) exhibited significantly higher CAF scores than the responder subgroup (n = 202) (p < 2.2e-16; **Figure 6C**). Low-CAF-risk patients (144/347) displayed higher immunotherapy sensitivity and lower TIDE scores than high-CAF-risk patients (58/347) (p < 0.001; **Figures 6D, E**). In GSE47994, the non-responder subgroup (n = 63) also had a significantly higher CAF score than the responder subgroup (n = 38) (p = 1.9e-7; **Figure 6G**). Low-CAF-risk patients (31/54) exhibited higher immunotherapy sensitivity and lower TIDE scores than high-CAF-risk patients (7/47) (p < 0.001; **Figures 6H, I**). The AUC values of 0.711 in TCGA-BRCA (**Figure 6F**) and 0.811 in GSE47994 (**Figure 6J**) indicate the excellent performance of the CAF model for predicting immunotherapy response.

### 3.7 GSEA of the five-gene CAF signature

**Figure 7:**
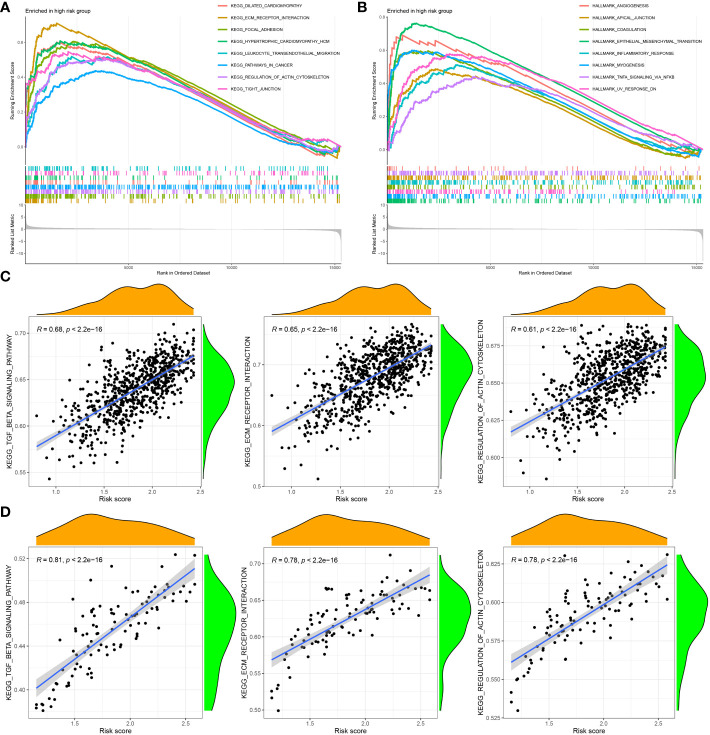
Gene set enrichment analysis (GSEA) of KEGG **(A)** and hallmark **(B)** gene sets between high‐and low‐CAF risk groups. **(C, D)** ssGSEA results showed CAF risk score was positively correlated with ECM receptor interaction, regulation of actin cytoskeleton, and TGF-β signaling pathway enrichment scores in both TCGA-BRCA **(C)** and GSE47994 **(D)**.

To investigate the functional enrichment of the CAF signature, GSEA was conducted on the TCGA-BRCA dataset to compare the high- and low-CAF-risk groups. The analysis revealed significant enrichment in KEGG signaling pathways associated with ECM receptor interaction, focal adhesion, pathways in cancer, regulation of actin cytoskeleton, and tight junction in the high-CAF-risk group (**Figure 7A**). Moreover, the genes in the high-CAF-risk group were significantly enriched in Hallmarker gene sets related to angiogenesis, apical junction, coagulation, epithelial-mesenchymal transition (EMT), and inflammatory response (**Figure 7B**). Additionally, ssGSEA results indicated that the CAF risk score was positively correlated with enrichment scores for ECM receptor interaction, regulation of actin cytoskeleton, and TGF-β signaling pathway in both TCGA-BRCA (**Figure 7C**) and GSE47994 (**Figure 7D**).

### 3.8 Cross-dataset validation of important genes in CCLE and HPA

**Figure 8:**
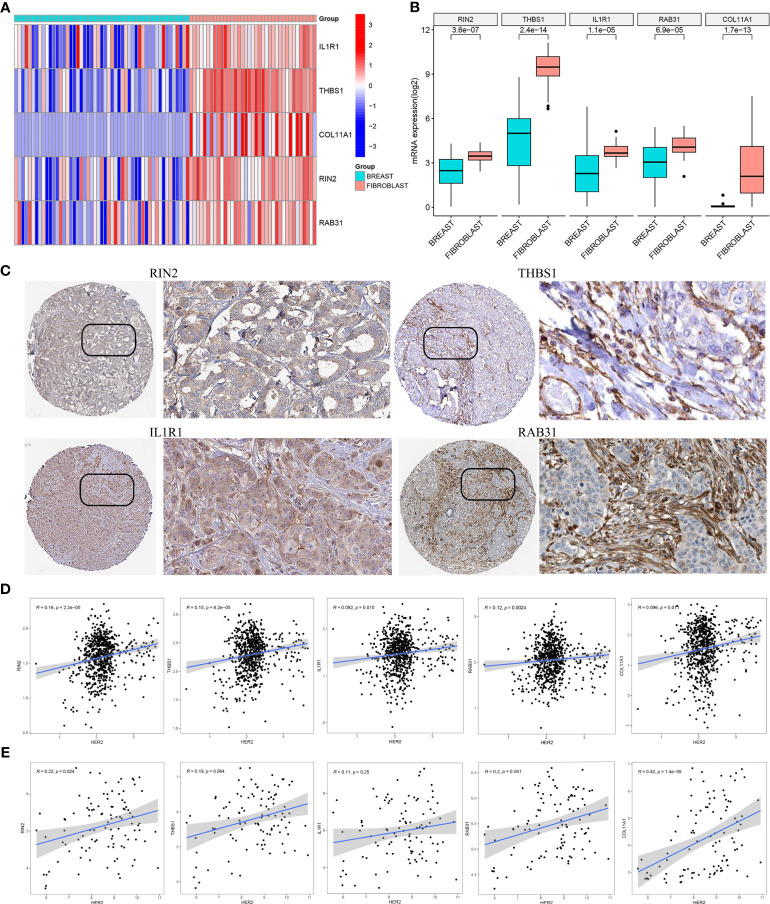
**(A, B)** The mRNA expression levels of the five CAF genes in the fibroblasts and breast cancer cell lines were illustrated in the heat map **(A)** and compared by Wilcoxon analysis **(B)**. **(C)** Protein expressions of RIN2, THBS1, IL1R1 and RAB31 in breast cancer specimens from the Human Protein Atlas database. Correlation between five hub genes and HER2 in TCGA-BRCA **(D)** and GSE47994 **(E)**.

Based on the CCLE database, the mRNA expressions of the five hub genes (RIN2, THBS1, IL1R1, RAB31, COL11A1) were verified to be higher in fibroblast cell lines than those in BC cell lines (Wilcoxon test, all p < 0.001; **Figures 8A, B**). Additionally, to determine the protein expression characteristics of these CAF signature genes, the IHC images from the HPA database were analyzed. The data demonstrated that these proteins (RIN2, THBS1, IL1R1 and RAB31) were deeply stained in BC stroma (**Figure 8C**). These verifications suggest that these genes might be CAF-specific markers.

### 3.9 Correlation between hub genes and HER2 in LBC

It is well known that the HER2 gene plays an important role in breast cancer, and overexpression or amplification of HER2 can lead to excess HER2 protein on the surface of breast cancer cells, leading to uncontrolled cell growth, tumor development and progression. To evaluate the correlation between the expression of the five hub genes (RIN2, THBS1, IL1R1, RAB31, COL11A1) and HER2 gene expression, we analyzed the gene expression data from TCGA-BRCA (**Figure 8D**) and GSE47994 (**Figure 8E**). Pearson correlation coefficient analysis revealed a significant positive correlation between the expression levels of these genes and HER2 gene expression (p < 0.05), with the exception of THBS1 and IL1R1 in GSE47994.

## 4 Discussion

Breast cancer is a complex and heterogeneous disease, consisting of multiple subtypes with distinct molecular and clinical characteristics (52). LBC is one of the most common subtypes, and some patients develop drug resistance and distant metastasis, and the prognosis of these patients is poor (4). While the molecular mechanisms underlying LBC development and progression have been extensively studied, the role of CAF in this subtype remains unclear. CAFs are a key component of the tumor microenvironment and have been shown to play a critical role in promoting tumor growth and progression, including in LBC (14, 19). Consistently, we observed that higher CAF and stromal scores were associated with worse OS after initial treatment in LBC. Therefore, identifying CAF-related factors and developing a CAF-related classifier for predicting prognosis and therapeutic effects in LBC is of great significance. This is the first study utilizing WGCNA and multiple computational algorithms to uncover mutual co-expression networks between CAF and stromal components in two LBC cohorts: TCGA-BRCA and GSE47994. Through the application of univariate Cox and LASSO regression algorithms, a five-gene prognostic model for CAF (comprising RIN2, THBS1, IL1R1, RAB31, and COL11A1) was developed and subsequently validated. We found that LBC patients with a low CAF risk (using the median CAF risk score of 1.85 in the training set as a threshold) may benefit from a variety of antineoplastic agents, such as Axitinib, Docetaxel, Fulvestrant, Lapatinib, Palbociclib, Ribociclib, Tamoxifen, and others, indicating that high CAF infiltration may contribute to these drugs resistance. On the other hand, LBC patients with a high CAF risk may be more responsive to treatments such as Alpelisib, Epirubicin, and dasatinib. We also utilized the TIDE online algorithm and observed a strong correlation between lower CAF risk scores and improved immunotherapeutic response in LBC patients. However, Further experiments are required to elucidate the interplay between CAFs and Immunotherapy. It’s worth noting that the TIDE algorithm primarily predicts the responses to anti-PD1 and anti-CTLA4 treatments in tumor patients, thus making pembrolizumab a more suitable candidate for follow-up studies.

To ensure the robustness of our model and avoid over-fitting, we employed four bioinformatics methods to quantify CAF infiltrations in LBC. We used the EPIC method for model construction and xCell, MCP-counter, and TIDE methods for correlation verification. Our results demonstrated a strong correlation between our model and CAF infiltrations, as well as CAF markers. Furthermore, based on analysis of the CCLE and HPA databases, we identified five genes as CAF-specific markers for LBC, with significantly higher expression observed in fibroblast cell lines and stromal parts of LBC. These findings further support the accuracy of our model in assessing CAF infiltration levels in LBC.

To investigate biological pathways associated with CAF risk in LBC, we performed GSEA analysis on TCGA-BRCA and GSE47994 dataset. GSEA revealed that ECM receptor interaction, focal adhesion, pathways in cancer, regulation of actin cytoskeleton and tight junction were highly and significantly enriched in the high–CAF-risk group; ssGSEA results also showed that the CAF risk score was positively correlated with ECM receptor interaction, regulation of actin cytoskeleton, and TGF-β signaling pathway enrichment scores in both two cohorts. CAFs play a crucial role in cancer progression by altering the extracellular matrix (ECM) composition and promoting cancer cell invasion and metastasis through the interaction of ECM proteins with specific receptors on the surface of cells (53–55). Furthermore, CAFs secrete fibronectin, which activates integrin receptors on cancer cells, triggering signaling pathways that enhance cancer cell proliferation, survival, and migration (56, 57). In addition, CAFs regulate the actin cytoskeleton of cancer cells, facilitating their ability to invade and migrate, by secreting growth factors such as TGF-β that promote the formation of stress fibers essential for cell migration and invasion (58, 59).

With respect to the five identified markers in the model, RIN2 is a gene that encodes a protein that interacts with Ras and Rab proteins, which are involved in cell signaling and membrane trafficking (60, 61). Biallelic defects in RIN2 are associated with MACS syndrome, a condition characterized by macrocephaly, alopecia, cutis laxa, and scoliosis, as well as with RIN2 syndrome, a related connective tissue disorder presenting similar symptoms (60, 62). Chiara Sandri et al. identified the Ras and Rab5 interacting protein RIN2 as a key effector in endothelial cells that interacts with R-Ras and mediates the pro-adhesion and tumor angiogenic activities of R-Ras (63). Furthermore, RIN2 has been identified as a signature gene that can be used to evaluate the clinical prognosis of patients with colorectal cancer, enabling more personalized diagnosis and treatment of the disease (64). THBS1 is a gene that encodes thrombospondin 1, a protein that is involved in cell adhesion, angiogenesis and inflammation (65). Previous studies have shown that THBS1 is highly expressed in gastric cancer (66), breast cancer (67), melanoma (68) and oral squamous carcinoma (69), promoting tumor cell adhesion, proliferation, apoptosis, invasion and metastasis. THBS1 can modulate the invasion and migration of BC cells by affecting the TME, especially the CAFs (70). We observed that high-CAF-risk group LBC patients were less sensitive to several drugs, including docetaxel, which is consistent with the finding that up-regulation of THBS1 following neoadjuvant chemotherapy containing docetaxel was associated with docetaxel chemotherapy resistance in breast cancer patients (71). Additionally, THBS1 can protect MCF-7 cells from docetaxel-induced apoptosis by activating the integrin β1/mTOR pathway (71). IL1R1 is expressed in various types of cancer cells and CAF, which are stromal cells that support tumor growth and survival (72–74). IL1R1 signaling can modulate various aspects of tumor biology, such as angiogenesis, invasion, metastasis, immunity and drug resistance (75). Rosamaria et al. found that the expression of IL1R1 is regulated by hypoxia-inducible factor 1α (HIF-1α) and G-protein estrogen receptor (GPER) in breast cancer cells and CAFs (72). Puran Zhang et al. found that high expression of IL1R1 in gastric cancer is indicative of poor prognosis and a poorer response to 5-fluorouracil-based adjuvant chemotherapy and immune checkpoint blockade (74). In addition, IL1R1 has been found to be upregulated in ALDH+ cells and plays a crucial role in driving cancer stem cell (CSC) activity, which can lead to resistance to adjuvant endocrine therapies, including tamoxifen and fulvestrant, in breast cancer and promote bone metastasis (76, 77). RAB31, a protein secreted by CAF, is associated with malignant behavior in breast (78), hepatocellular (79), gastric (80), and colorectal cancers (81). Studies have shown the expression levels of RAB31 may serve as a crucial regulator of the transition between invasiveness and proliferation of breast cancer cells (78, 82). Recent research has shown that RAB31 is capable of inhibiting the TGF-β pathway by decreasing TGFB1 mRNA and antigen levels, thereby exerting an impact on the migration, invasion, and apoptosis of breast cancer cells (82). Additionally, Rab31 mediates cisplatin resistance and metastasis in stomach adenocarcinoma *via* epithelial-mesenchymal transition pathway (83). COL11A1 is a gene that encodes for collagen type XI alpha 1, a protein that is part of the extracellular matrix (ECM) (84). Studies have shown that COL11A1 can activate CAFs by stimulating the TGF-β signaling pathway that regulates cell proliferation and differentiation (85, 86). COL11A1 can also promotes cancer cell migration, metastasis, and therapy resistance by activating multiple signaling pathways (84, 87). Notably, COL11A1 has been shown to induce chemoresistance to cisplatin and paclitaxel in ovarian cancer cells through the AKT and Twist1 pathways (88, 89). Moreover, it may promote tumor immune infiltration and lead to a poor prognosis in breast cancer patients (90). However, there is not much functional validation of the five genes involved in risk signatures in the CAFs of LBC, which requires us to conduct further experiments on the five CAF markers in the future to evaluate the invasion and metastasis, drug resistance and immunosuppression of LBC.

It is worth mentioning that, in the initial analysis, we conducted the same analysis in the TCGA-BRCA database for different subtypes of breast cancer and found no significant difference in survival when analyzing CAF infiltration and stromal score in triple-negative breast cancer and HER2-positive breast cancer. However, Pearson correlation coefficient analysis revealed a significant positive correlation between the expression levels of these genes and HER2 gene expression in LBC. Meanwhile, targeted immunotherapy targeting cancer-associated fibroblasts has been reported to overcome drug resistance in HER2+ breast cancer treatment (91). Therefore, more analytical methods and experiments are needed to investigate the potential relationship between HER2 gene and CAF signature genes.

In conclusion, our study identified a five-gene CAF signature for predicting prognosis and therapeutic responses in LBC. Our findings provide important insights into the role of CAFs in promoting tumor growth and progression and highlight the importance of developing combination therapies that target both CAFs and the immune system. Our study has important clinical implications for guiding tailored anti-CAF therapy in combination with immunotherapy for LBC patients. There are also some limitations to our study. First, our study is retrospective, and therefore, our findings should be validated in a prospective study. Second, we did not perform functional experiments to validate the role of the identified CAF markers in promoting tumor growth and progression in LBC. Future studies should investigate the molecular mechanisms underlying the identified CAF markers and develop targeted therapies that can inhibit CAFs and promote anti-tumor immune responses.

## Data availability statement

The original contributions presented in the study are included in the article/supplementary material. Further inquiries can be directed to the corresponding author.

## Author contributions

AX and X-NX contributed to the conception and design. AX and ZL extracted the data from the databases. AX, X-NX, and XH contributed to the data analysis and interpretation. AX and X-NX drafted the manuscript. XH and D-YF revised the manuscript. D-YF supervised the entire study. All authors contributed to the article and approved the submitted version.
